# The synthesis of well-defined poly(vinylbenzyl chloride)-grafted nanoparticles via RAFT polymerization

**DOI:** 10.3762/bjoc.9.139

**Published:** 2013-06-25

**Authors:** John Moraes, Kohji Ohno, Guillaume Gody, Thomas Maschmeyer, Sébastien Perrier

**Affiliations:** 1 Key Centre for Polymers & Colloids, School of Chemistry, The University of Sydney, NSW 2006, Australia; 2 Institute for Chemical Research, Kyoto University, Kyoto 611-0011, Japan; 3 Laboratory of Advanced Catalysis for Sustainability, School of Chemistry, The University of Sydney, NSW 2006, Australia

**Keywords:** core–shell particles, free radical, grafting, RAFT polymerization, silica

## Abstract

We describe the use of one of the most advanced radical polymerization techniques, the reversible addition fragmentation chain transfer (RAFT) process, to produce highly functional core–shell particles based on a silica core and a shell made of functional polymeric chains with very well controlled structure. The versatility of RAFT polymerization is illustrated by the control of the polymerization of vinylbenzyl chloride (VBC), a highly functional monomer, with the aim of designing silica core–poly(VBC) shell nanoparticles. Optimal conditions for the control of VBC polymerization by RAFT are first established, followed by the use of the “grafting from” method to yield polymeric brushes that form a well-defined shell surrounding the silica core. We obtain particles that are monodisperse in size, and we demonstrate that the exceptional control over their dimensions is achieved by careful tailoring the conditions of the radical polymerization.

## Introduction

The versatility of organic free radical chemistry in terms of functionality and reaction conditions makes it a technique of choice for the synthesis of functional polymeric materials. However, the lack of control over the chain length and chain end of the final polymeric material makes conventional radical processes unsuitable for specific targeted applications. The establishment in the 1990s of living radical polymerization (LRP, defined as reversible deactivation radical polymerization by the IUPAC), has dramatically changed the polymer-synthesis landscape allowing the easy production of well-defined polymeric materials of desired molecular weights with narrow dispersity (<1.5) and complex architectures (i.e., block copolymers) [1]. By exploiting a dormant state of the propagating radical of a growing chain, it is possible to limit the proportion of irreversibly terminated chains in a radical polymerization, and thus to control the final structure of the resulting polymeric chain. Among the many techniques of LRP reported to date, reversible addition–fragmentation chain transfer (RAFT) polymerization is one of the most versatile processes, both in terms of tolerance towards a wide range of monomer functionality and reaction conditions [2]. RAFT polymerization employs a chain transfer agent (CTA, or RAFT agent), which is reversibly transferred from one propagating chain to another in a degenerative process. The rapid exchange of the RAFT agent between propagating chains ensures that each chain grows simultaneously over the course of the polymerization and a final narrow molecular weight distribution of the polymer product ensues. Moreover, the molecular weight of the final material can be easily tuned depending on the amount of CTA initially introduced. This degenerative process is triggered by the presence of radicals, typically obtained from thermal or photoinitiators, the amount of which is kept low in comparison with the amount of CTA introduced (i.e., high ratio CTA/initiator) in order to minimise the fraction of dead chains produced. Therefore, it is commonly assumed that the ratio of monomer to RAFT agent gives the average degree of polymerization (DP, i.e., the number of monomers per chain) [3–4].

RAFT polymerization has been used to generate a very large range of materials ranging from polymeric architectures to nanomaterials and hybrid materials [5–9]. In particular, RAFT has had a major impact in addressing the challenge of preparing highly monodisperse core–shell nanoparticles, which hold great promise for a range of applications such as drug-delivery vectors or colloidal-crystal self-assemblies [10–11]. RAFT polymerization initiated from preformed inorganic nanoparticles enables the grafting of polymer shells from the particle surface and yields well-defined particles from a range of monomeric precursors [12]. While initial work focussed on common monomers such as styrene [13–15] and (meth)acrylates [14–16], several recent papers seek to extend this work to a greater variety of monomers [17–22]. One particular motivation has been the post-polymerization functionalization of the grafted chains to yield functional nanoparticles. Therefore, monomers that allow such post*-*polymerization functionalization are beginning to attract greater research attention [23–24].

4-Vinylbenzyl chloride (VBC) is one such monomer that offers ready post*-*polymerization functionalization through the pendant chloride group [25–31], which can readily undergo nucleophilic substitution [25–29] or be used as an initiating site for another LRP system, i.e., atom transfer radical polymerization (ATRP) [30–31]. It has, therefore, been used in a variety of systems as a precursor to glycopolymer stars [29], photo- and pH-responsive nanoparticles [30], nanofibres [28], comb, graft and star polymers [27], and triblock copolymers [26]. While there have been reports of the (co)polymerization of VBC by RAFT techniques [25–30,32], the polymerization of this highly versatile monomer onto solid scaffolds has, thus far, not been described. RAFT polymerization is an ideal radical process technique for VBC as side reactions (such as dissociation of the C–Cl bond, which would be expected if ATRP were used to polymerize VBC) can be avoided [27,32]. For the purposes of this study, high-molecular-weight chains (ca. 20 to 100 kg/mol) are of importance, as the ability to grow high-molecular-weight chains from the surface of the silica particles allows us to increase the number of functionalizable benzyl chloride groups present. Additionally, having a large amount of polymer grown from the particle will allow fine control over the effective diameter of the particle by merely tuning the polymerization conditions to dictate the size of the polymer shell. This cannot be achieved if low molecular weights are targeted, as their comparative contribution to the diameter of the particle is negligible.

**Scheme 1:**
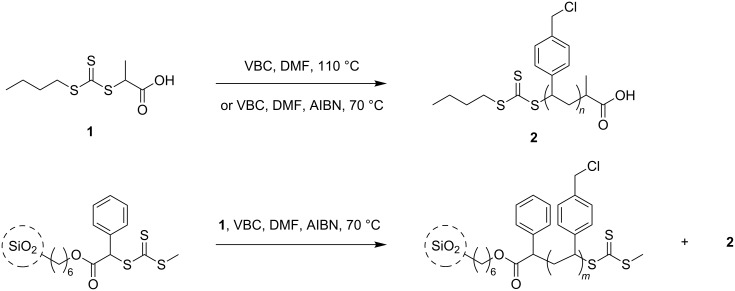
RAFT polymerization and silica-supported RAFT polymerization of vinylbenzyl chloride (VBC).

This manuscript focuses on two aspects of RAFT polymerization. In the first instance, we explore the use of RAFT polymerization with either thermal autoinitiation of VBC or thermal initiation by an azoinitiator to achieve a well-controlled polymerization of the monomer in solution (Scheme 1). We then use the latter approach to form well-defined core–shell nanoparticles wherein the size of the polymer shell can be varied by changing the degree of polymerization of the grafted polymer chains.

## Results and Discussion

Previous literature on the RAFT-mediated polymerization of vinylbenzyl chloride has utilized either thermal autoinitiation [29], or azoinitiators [25–28,32] and photoinitiators [33]. Of these investigations only two were concerned with polymers greater than 20 kg/mol, and hence, our studies focussed on the RAFT polymerization of VBC using either thermal autoinitiation or an azoinitiator [26,29]. The former method is reported to yield faster kinetics due to the higher *k*_p_ at elevated temperatures [29] and, thus, initial experiments in this study were conducted at 110 °C in the presence of the RAFT agent 2-(butylthiocarbonothioylthio)propionic acid (PABTC) without any additional initiator. In addition to targeting a high-molecular-weight polymer by choosing a high degree of polymerization (DP), we also prepared polymers of lower molecular weights (DP 100) to serve as a point of comparison.

**Figure 1:**
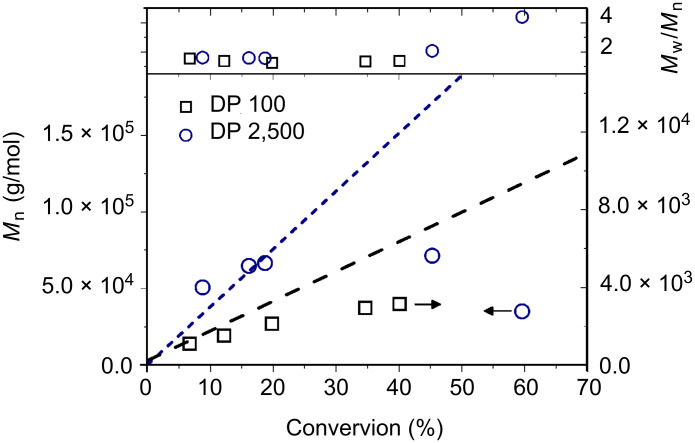
Evolution of number-average molecular weight (*M*_n_) and *M*_w_/*M*_n_ values of the poly(VBC) chains obtained by RAFT polymerization with PABTC as CTA and thermal autoinitiation. Dashed lines depict the theoretical molecular weights obtained from Equation 1 without taking into account the thermally initiated chains.

The two polymerizations showed conventional kinetics features for a radical polymerization, i.e., increase of the monomer conversion with increasing reaction time and linear semilog kinetics plots (Supporting Information File 1, Figure S1), although significant rate retardation, typically observed in RAFT polymerization [34], was noted for lower DP targeted. Size-exclusion chromatography (SEC) analysis of the polymer chains showed that for both DPs targeted, a linear increase of the molecular weight was noted with increasing conversions of up to ca. 20%. After this point, however, the molecular weights taper off or steadily decrease for polymers of DP 100 and DP 2,500, respectively (Figure 1). We hypothesise that this negative deviation of the experimental molecular weight derived from the large number of thermally initiated chains in solution. In fact, in the case of thermal autoinitiation, since the monomer plays also the role of the initiator, the concentration of initiator would be intimately linked to the monomer concentration. Thus, when high DP are targeted (i.e., low [CTA]_0_), it is expected that the concentration of monomer-initiated chains (i.e., VBC-derived chains) would greatly outnumber the chains initiated by the R-group of the RAFT agent (i.e., CTA-derived chains). If the number of thermally initiated chains is not negligible in comparison with the number of CTA-derived chains (typically, <10% of the total number of chains is considered negligible) a negative deviation of the theoretical molecular weight is expected, as seen in Equation 1 where [VBC]_0_ and [PABTC]_0_ are the initial concentration in VBC and RAFT agent, [thermally initiated chains] is the concentration of generated VBC initiator species, C is the monomer conversion, and *M*_(VBC)_ and *M*_(PABTC)_ are the molar masses of monomer and chain transfer agent, respectively.

[1]
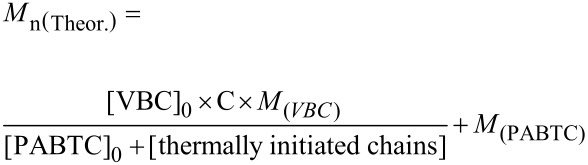


This important feature would also explain the poor uniformity of the final material obtained (cf. the high *M*_w_/*M*_n_ values in Figure 1) as the final number of dead chains (chains not possessing a trithiocarbonate, from the RAFT agent ω-end group and thereby nonliving) is related to the total number of VBC-initiated chains. Therefore, when high DPs are targeted, the fraction of VBC-initiated chains (i.e., dead polymer chains) is expected to be very large. It is worth noting that this negative deviation of the experimental molecular weight with the monomer conversion together with high *M*_w_/*M*_n_ values when high DPs are targeted has also been previously observed by Chen et al. who prepared star polymers of VBC using thermal autoinitiation [29].

**Figure 2:**
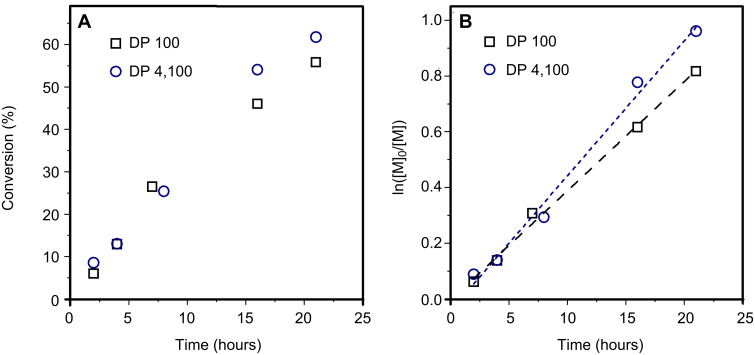
Conversion and ln([M]_0_/[M]) versus time for AIBN-initiated, PABTC-mediated polymerization of VBC at DP 100 (squares) and DP 4,100 (circles): (A) monomer conversion versus reaction time; (B) semilog kinetics plots with dashed lines indicating linear fits of the data (DP 100, long dashes; DP 4,100, short dashes).

Since thermal autoinitiation resulted in poor control over the polymer chains, we next investigated the use of an azoinitiator, 2,2′-azobis(2-methylpropionitrile) (AIBN). For these experiments, two DPs (100 and 4,100) were targeted, keeping the initial concentration of AIBN and monomer constant so as to obtain similar kinetics for each experiment. As seen in Figure 2, the polymerizations in each case proceeded at almost identical rates (although Figure 2B shows slight retardation at DP 100, as expected for RAFT polymerization targeting lower DPs, see above), irrespective of the DP, while similar linear semilog kinetics plots for each experiment suggest that the radical flux is constant over the time scale of the experiments.

For both polymerizations, the molecular weights of the poly(VBC) chains are close to the theoretical molecular weights (see Table 1). There were two important considerations associated with this finding: (1) the experimental molecular weights of the polymers (*M*_n(Exp.)_) were determined by using a SEC system calibrated with narrow-molecular-weight poly(styrene) standards. Since this system results in *M*_n(Exp.)_ values relative to poly(styrene), these *M*_n(Exp.)_ were corrected by taking into account the difference in molecular weight between VBC and styrene (see Table 1, footnote b). (2) In order to accurately determine the theoretical molecular weight for each experiment, it is crucial to also take into account the concentration of AIBN-initiated chains generated by the decomposition of AIBN throughout the polymerization (see Equation 2 where *f* is the initiator efficiency (assumed to be 0.5 for AIBN), the term “2” means that 1 molecule of azoinitiator gives two primary radicals, *k*_d_ is the dissociation constant of AIBN at 60 °C (9.8 × 10^−6^ s^−1^), *t* is the time in seconds, and *f*_c_ is the coupling constant (*f*_c_ = 1 for 100% termination by combination and *f*_c_ = 0 for 100% termination by disproportionation), assumed to be 1 in this case since poly(VBC) and poly(styrene) are considered to terminate primarily by combination). This is especially true in the case when high DPs are targeted (for instance 4,100) where the ratio of AIBN to PABTC is 5:1, which results in a substantial proportion (71%) of AIBN-derived chains after 21 h.

**Table 1:** Characteristics of polymers produced by the AIBN-initiated, PABTC-mediated polymerization of VBC at 60 °C.

Expt. No.	Time (h)	Conversion^a^	*M*_n_ (Theory) (g/mol)	*M*_n_ (Exp.) (g/mol)^b^	*M*_w_/*M*_n_	AIBN-initiated chains (%)^c^

1^d^	2	6%	2,000	2,300	1.36	1%
2^d^	4	13%	2,400	2,700	1.42	1%
3^d^	7	26%	3,600	4,000	1.39	2%
4^d^	16	46%	6,000	6,500	1.32	4%
5^d^	21	56%	7,800	8,400	1.27	5%
6^e^	2	8%	40,800	42,800	1.84	24%
7^e^	4	13%	50,900	50,000	1.95	38%
8^e^	8	25%	74,700	72,900	1.77	53%
9^e^	16	54%	113,200	98,800	1.91	67%
10^e^	21	62%	113,300	110,200	1.97	71%

^a^Determined by ^1^H NMR. ^b^Determined by SEC: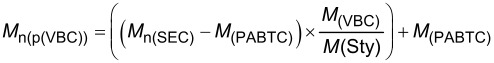. ^c^From the ratio of AIBN-initiated chains (calculated in a similar manner as in Equation 2) to total chains. ^d^Polymerizations carried out at 60 °C in DMF (10 wt %) and [VBC]_0_/[PABTC]_0_/[AIBN]_0_ = 100/1/0.1. ^e^Polymerizations carried out at 60 °C in DMF (9 wt %) and [VBC]_0_/[PABTC]_0_/[AIBN]_0_ = 4,100/1/5.

[2]
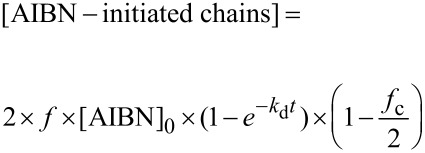


As seen in Table 1, the contribution of the AIBN-initiated chains to the total number of chains becomes significant at higher DPs and explains the negative deviation of *M*_n(Exp.)_ under these conditions. The presence of these AIBN-initiated chains also adversely affects the *M*_w_/*M*_n_ values of the polymers at DP 4,100, which are consistently higher than those at DP 100 (compare experiments 6–10 with experiments 1–5 in Table 1). However, this is an unavoidable consequence of RAFT polymerization under these conditions, when targeting such extremely high DPs. Nonetheless, since a predictable increase in *M*_n_ with conversion was demonstrated (a key requirement for the controlled synthesis of core–shell particles), we proceed to undertake the polymerization in the presence of silica-supported RAFT agents.

In the preparation of the silica–polymer hybrid particles, our aim was to form polymer brushes on the surface of the particles. Thus, the so-called “grafting from” approach, where the R-group of the RAFT agent is attached to the silica particle [12,35], was used to obtain a high grafting density [36]. The sulfur content of the particles (hereby SiP–RAFT) was determined by elemental microanalysis, and the grafting density of RAFT–agents on the surface of the particles was calculated to be 0.4 groups·nm^−2^ (see Supporting Information File 1, Equation S1). SiP–RAFT particles were added into the polymerization media such that the particles accounted for 1 wt % of the total mass of the reactants. In addition to the silica-supported RAFT agent, free PABTC was also added to the system in order to maintain control over the polymerization, as previously described [37]. Using the grafting density of the SiP–RAFT, we calculated that the tethered RAFT agent accounts for ca. 10% of the total RAFT agent in the reaction. Thus, two distinct types of RAFT-mediated chains are present in the solution. The first type is derived from the free RAFT agent, while the second type is anchored to the silica surface.

**Figure 3:**
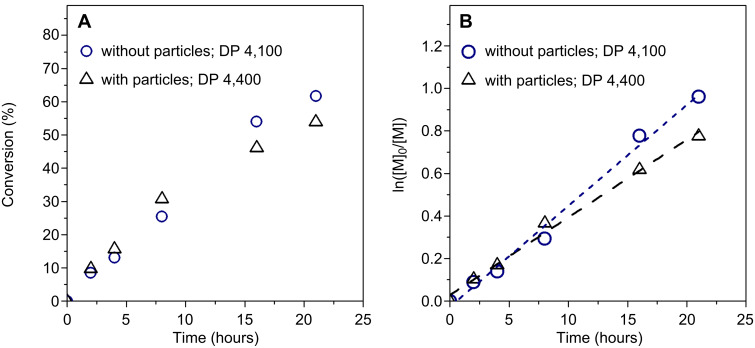
AIBN-initiated, PABTC-mediated polymerization of VBC with (triangles, DP 4,400) and without (circles, DP 4,100) SiP–RAFT particles: (A) monomer conversion versus reaction time; (B) pseudo-first order plots with dashed lines indicating linear fits of the data (long dashes, with particles, DP 4,400; short dashes, without particles, DP 4,100).

The silica-supported RAFT polymerization was performed at a DP of 4,400 and an initiator concentration of 7.22 × 10^−3^ mol·L^−1^ (CTA/AIBN ratio of 1/5), conditions analogous to experiments 6–10 in Table 1 above. As seen in Figure 3, the addition of the SiP–RAFT particles to the polymerization media does not have any deleterious effect on the polymerization, and similar kinetics are observed in the experiments with and without particles, as expected since SiP–RAFT only accounts for ca. 10% of the total RAFT agent in the reaction.

**Figure 4:**
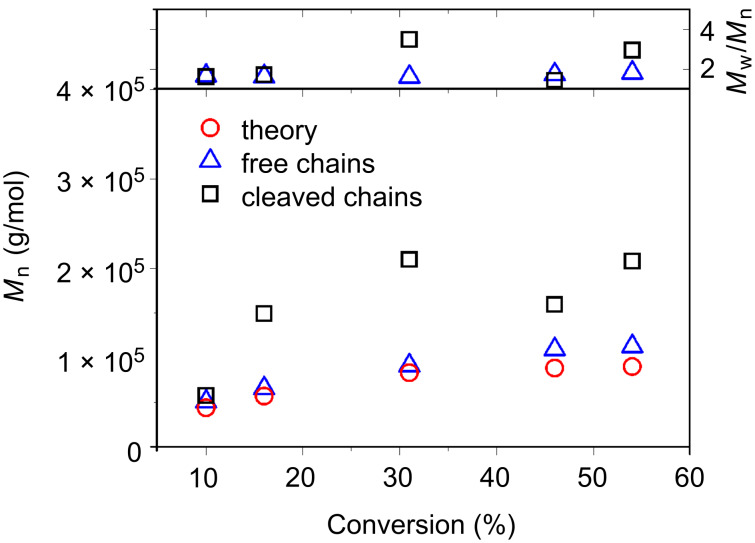
Evolution of molecular weights of free and grafted poly(VBC) chains with conversion.

The free polymer chains are readily separated from the silica particles by dilution of the polymerization mixture with THF and subsequent centrifugation. SEC analysis shows that there is a very close adherence of the molecular weight of the free chains to the theoretically expected values at each step of the polymerization (Figure 4) and the chains maintained a monomodal size distribution throughout the polymerization (see Supporting Information File 1, Figure S2). Grafted chains were liberated from the particles (before being subjected to SEC analysis) by using hydrofluoric acid (HF) to destroy the silica core by etching. These chains show higher *M*_n(Exp.)_ than the theory and higher *M*_w_/*M*_n_ values than for the free polymer chains, particularly at higher conversions. This observation can be attributed either to a poorer control over the RAFT process, for instance the possible occurrence of branching due to chain transfer between grafted polymeric chains and other side reactions occurring during the RAFT process and enhanced by the high local concentration of grafted chains [34,38–39], or the result of the harsh conditions used to etch the silica, which may affect the polymeric chains. Indeed, there were several difficulties encountered during the etching experiments with high pressures being noted in the SEC system when eluting samples. In addition, in contrast to the free polymer chains some bimodality was observed in the etched polymer chains (see Supporting Information File 1, Figure S3 cf*.* Figure S2). No conclusive elucidation of any degradation to degrafted chains was possible, as the amount of material recovered was insufficient for ^1^H NMR analysis. The high pressures in the SEC were particularly prevalent in the two samples taken later in the polymerization (at 16 hours and 21 hours). Thus, the SEC data for these two samples may be underestimated (i.e., the longest polymer chains may have been removed during the filtration). What is evident, however, is that in nearly every sample, the molecular weight of the grafted polymer is higher than that of the free polymer. This is similar to what was previously observed in the thermally autoinitiated SiP–RAFT-mediated polymerization of styrene, since the molecular weight of the grafted chains is not affected by the thermally initiated free chains, the presence of which contributes to lower the molecular weight of the nongrafted chains [36]. A thorough analysis of the hybrid nanoparticles at each of the kinetic points was then carried out.

**Figure 5:**
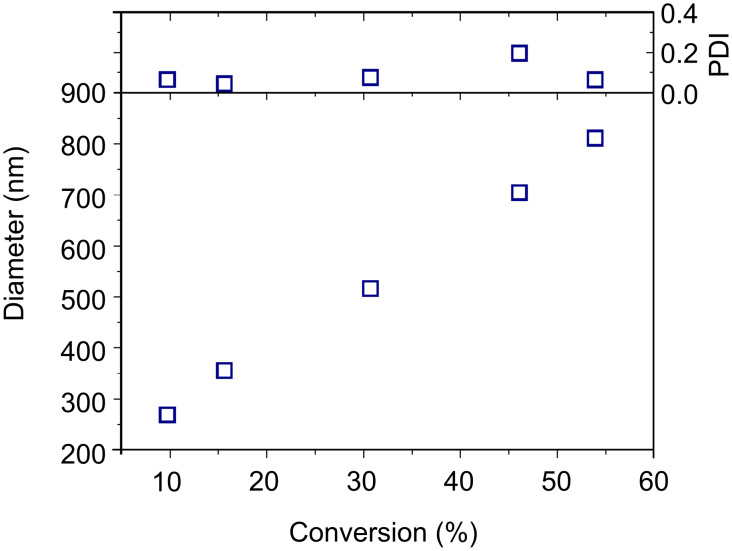
Plot of the average diameter and PDI of particles recovered from silica-supported RAFT polymerization of VBC.

The particles were washed by repeated centrifugation–redispersion cycles in THF in order to completely remove free polymer chains adsorbed onto the particles. The particles (henceforth SiP–p(VBC) particles) were studied by dynamic light scattering (DLS), which showed a monomodal peak indicating well-defined particles with no aggregation (Supporting Information File 1, Figure S4). There is a clear increase in the diameters of the particles as the reaction proceeds, indicating a growth of the polymer shell surrounding the silica core. Plotting the average diameter and polydispersity index (PDI) of the particles against monomer conversion (Figure 5), shows an increase in particle size with increasing conversion. This continues past the first three data points in stark contrast to the *M*_n_ of the polymer chains degrafted from the particles. This strongly suggests that the grafted chains do indeed continue to increase in size, despite the plateau observed in the SEC (which could be an artefact of the SEC analysis and the loss of higher-molecular-weight chains on the SEC filters, thereby resulting in the high system pressures mentioned previously). Alternatively, assuming the SEC analysis is an accurate depiction of the polymer chains, it is possible that even though the growth of the polymer chains slows down at higher conversions, the particles in solution continue to increase in size due to the solvent swelling the grafted polymer chains, thus increasing the apparent particle diameter.

It is also noteworthy that the PDI of the particles remains low throughout the reaction (PDI < 0.2), in contrast to the relatively high PDIs obtained for both the grafted and free polymeric chains from this reaction (Figure 4). This observation shows that despite the high *M*_w_/*M*_n_ values for the poly(VBC) chains and the number of initiator-derived chains in the system, a very well controlled growth of particles is achievable and the size of the hybrid particle can be dictated as desired.

Thermogravimetric analysis (TGA) of the hybrid nanoparticles recovered from the reaction showed a steady increase in mass loss with increasing monomer conversion (Supporting Information File 1, Figure S5). Plotting the mass lost against conversion shows an almost linear trend indicating that the addition of polymer to the silica particles proceeds in a controlled manner, thus allowing precise incorporation of the required amount of VBC onto the silica particle. The mass loss on TGA, accompanied with the *M*_n_ of the (cleaved) chains measured by SEC allows calculation of the grafting density of the particles (See Supporting Information File 1, Equation S2). As seen in Supporting Information File 1, Figure S5, this remains nearly constant throughout the polymerization with an average value of 0.11 chains/nm^2^ (compared to 0.18 chains/nm^2^ if the *M*_n_ of the free chains is used for calculation).

**Figure 6:**
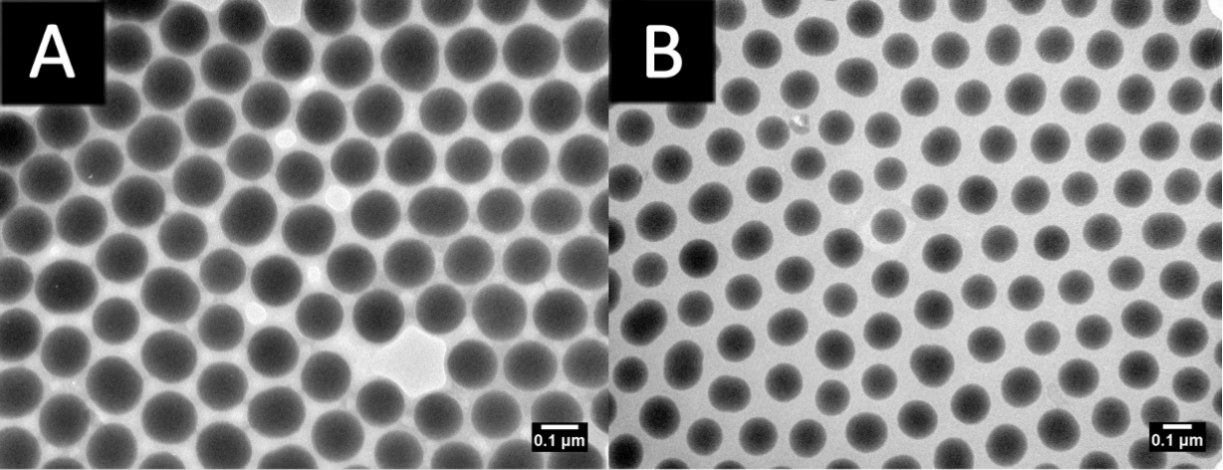
TEM micrographs of particles (A) after 2 hours and (B) after 21 hours of VBC polymerization. Scale bars represent 0.1 μm.

Transmission electron microscope (TEM) analysis of the particles recovered from the reaction shows that as the reaction proceeds, the polymer shell around the particles increases in size (Figure 6). The polymer shell is visible as the dark grey region between the particles, and it increases in size from 10% conversion (57,600 g/mol, grafted polymer) to 54% conversion (208,000 g/mol, grafted polymer). TEM samples of particles recovered from intermediate stages of the polymerization are included in Supporting Information File 1, Figure S6. It should be noted that TEM images of the particles show the average diameter of the particles to be smaller than that measured by DLS. We ascribe this to the fact that the polymer shell on the particles in DLS analysis is measured in a swollen state with a presumably fully extended chain, whereas the shell visible in the TEM images is desolvated and consequently appears in a shrunken state. We consider the size obtained by DLS as a more accurate depiction of the particles, as this technique assays a much greater number of particles and provides fuller information regarding the distribution of particle sizes in the samples. The pervasive presence of these polymer shells, keeping the particles from aggregating, is evidenced by the uniform distance between the particles. Thus, well-defined core–shell nanoparticles of tunable sizes are readily available using surface-initiated RAFT of VBC.

## Conclusion

We have demonstrated the controlled polymerization and grafting onto silica nanoparticles of 4-vinylbenzyl chloride using RAFT polymerization. Whilst thermal autoinitiation of VBC does not lead to well-controlled molecular weight at high conversions, the control is improved by using AIBN as initiator and lower temperatures for DPs around 100, whilst targeting DPs of an order of magnitude higher in similar conditions lead to poorer molecular-weight control, mainly due to the large contribution of terminated polymeric chains. When polymerising VBC in the presence of SiP–RAFT, using AIBN as an azoinitiator in the reaction medium resulted in a linear evolution of the final particle sizes with conversion. This allowed the desired particles size to be reliably synthesised with a high degree of monodispersity. Indeed, the particles recovered show monomodal particle-size distribution and very low dispersities. Their uniformity results in the formation of well-ordered films, showing long-range two-dimensional order.

## Supporting Information

File 1 Experimental procedures, equations, kinetic plots, SEC data, light scattering data, thermolysis data and TEM images.
