# Supplementary material for: The synthesis of well-defined poly(vinylbenzyl chloride)-grafted nanoparticles via RAFT polymerization
Source: Beilstein J Org Chem. 2013 Jun 25;9:1226–34. doi: 10.3762/bjoc.9.139 (PMC3701384; doi:10.3762/bjoc.9.139)
Supplement: File 1 — Experimental procedures, equations, kinetic plots, SEC data, light scattering data, thermolysis data and TEM images. [file Beilstein_J_Org_Chem-09-1226-s001.pdf]

Supporting Information
for
The synthesis of well-defined poly(vinylbenzyl chloride)-grafted nanoparticles via RAFT polymerization

John Moraes[1], Kohji Ohno[2], Guillaume Gody[1], Thomas Maschmeyer[3] and Sébastien Perrier[1*]

Address: [1]Key Centre for Polymers & Colloids, School of Chemistry, The University of Sydney, NSW 2006, Australia, [2]Institute for Chemical Research, Kyoto University, Kyoto 611-0011, Japan and [3]Laboratory of Advanced Catalysis for Sustainability, School of Chemistry, The University of Sydney, NSW 2006, Australia

Email: Sébastien Perrier* - sebastien.perrier@sydney.edu.au

*Corresponding author

Experimental procedures, equations, kinetic plots, SEC data, light scattering data, thermolysis data and TEM images.

Experimental

Materials

All reagents were purchased from Sigma-Aldrich at the highest purity and used as received unless otherwise stated. 4-Vinylbenzyl chloride (VBC) was passed through a column of activated neutral alumina in order to remove the inhibitor before use. Silica particles (SEAHOSTER KE-E10, average diameter = 130 nm, 20 wt % suspension of SiP in ethylene glycol) were kindly donated by Nippon Shokubai Co., Ltd., Osaka, Japan.

2-(Butylthiocarbonothioylthio)propionic acid (PABTC) was kindly provided by Dr. Algi Serelis of Dulux Group Australia. AIBN was recrystallised from methanol before use and stored at 4 °C. All polymerizations were carried out under a N_2 atmosphere.

Equipment

Molar mass distributions were measured using SEC on a Shimadzu LC-10AT liquid chromatograph system with a Polymer Laboratories PLgel 5 μM guard column and two PLgel Mixed-B columns using THF at 1.0 mL/min as the eluent at 40 °C. The system was equipped with a Shimadzu RID-10A differential refractive index detector and a Shimadzu SPD-10A UV-Vis detector. ^{1}H-NMR spectra were acquired on a Bruker 300 or 200 MHz in deuterated chloroform.

Transmission electron microscope (TEM) images were obtained on a JEOL 1400 120 kV TEM with a LaB_6 filament running Gatan Digital Micrograph software. Dynamic Light Scattering (DLS) was conducted on a Malvern High Performance Particle Sizer running Dispersion Technology Software (Version 4.00). Thermogravimetric analysis (TGA) was conducted on a TA Instruments Hi-res TGA 2950 instrument using Thermal Advantage v1.1A software. The instrument was continuously purged with N_2 gas. Samples were heated to 100 °C and equilibrated at that temperature to remove residual water prior to analysis. Data was analysed using TA Instruments' Universal Analysis 2000 software v4.2E.

Thermally auto-initiated, RAFT-mediated polymerization of VBC

In a typical reaction, to 4-vinylbenzyl chloride (3.64 g, 23.9 mmol) was added DMF (486 mg, 3.18 mmol) and PABTC (2.3 mg, 9.65 μmol). The constituents were mixed together with a vortex mixer and divided into separate reaction vials. The vials were sealed with a rubber septum and deoxygenated by sparging with nitrogen before beginning RAFT polymerization by immersing the vials in an oil-bath thermostatted at 110 °C. The reactions were quenched by exposing the samples to atmosphere and rapidly cooling them. The samples were diluted with deuterated chloroform for ^{1}H NMR analysis. Samples were diluted in SEC sample preparation solution for SEC analysis.

AIBN-initiated, RAFT-mediated polymerization of VBC

In a typical reaction, to 4-vinylbenzyl chloride (3.61 g, 23.7 mmol) was added AIBN (3.9 mg, 24 μmol), DMF (405 mg, 2.65 mmol) and PABTC (55.8 mg, 235 μmol). The constituents were mixed together with a vortex mixer, divided into separate reaction vials. The vials were sealed with a rubber septum and deoxygenated by sparging with nitrogen before beginning RAFT polymerization by immersing the vials in an oil-bath thermostated at 60 °C. The reactions were quenched and analysed as described above.

Silica-supported, AIBN-initiated, RAFT-mediated polymerization of VBC

In a typical reaction, to 4-vinylbenzyl chloride (8.999 g, 58.97 mmol) was added a suspension of the SiP–RAFT particles in DMF (7.553 mg of a 13.34 wt % solution such that there were 100.7 mg of particles in 4.278 mmol of DMF), DMF (264.2 mg, 1.727 mmol), AIBN (11.0 mg, 67.0 μmol) and PABTC (3.2 mg, 13 μmol). The constituents were mixed together with a vortex mixer and divided into separate reaction vials equipped with magnetic stirring fleas. The vials were sealed with a rubber septum and deoxygenated by sparging with nitrogen before beginning RAFT polymerization by immersing the vials in an oil-bath thermostated at 60 °C. The reactions were quenched by exposing the samples to atmosphere and rapidly cooling them. The samples were diluted with deuterated chloroform for ^{1}H NMR analysis. Samples were diluted in SEC sample preparation solution and centrifuged to remove the particles from the suspension for SEC analysis. The particles were washed by at least three centrifugation–redispersion cycles in THF before TGA, DLS and TEM analyses.

Cleaving poly(VBC) chains from silica nanoparticles

SiP–p(VBC)-RAFT particles were dispersed in toluene such that 10 mg of the hybrid nanoparticles were present in 5 mL of toluene. This suspension was added to 5 mL of a 10% v/v solution of HF in a plastic bottle containing a magnetic stirring flea. The resulting biphasic mixture was stirred vigorously for three hours at room temperature before the HF was neutralised by the addition of a 30 mL of a saturated aqueous solution of boric acid. The organic layer was then extracted with 20 mL of brine and 20 mL of water before being dried over calcium chloride. The solvent was then removed on a rotary evaporator to recover the polymer.

Calculating the density of RAFT agents on silica particles

The number of RAFT groups per nm^2 was calculated by analysis of the sulfur content of the particles by elemental microanalysis (conducted by the Microanalytical Facility at The Research School of Chemistry, Canberra Australia). The density of groups was calculated using Equation S1 where d is the grafting density, $w_{(S)}$ is the percent mass of sulfur, $M_{(S)}$ is the molar mass of sulfur, A_v is Avogadro's number, $\rho_{(SiP)}$ is the density of silica particles (1.9 x 10^{-21} $g{\cdot}nm^{-3}$), $V_{(SiP)}$ is the volume, and $SA_{(SiP)}$ is the surface area of a 130 nm silica particle.

Equation S1:

$$d = \frac{\frac{w_{(S)} \times M_{(S)}}{100 \times 3} \times A_v}{\frac{\rho_{(SiP)}}{V_{(SiP)}} \times SA_{(SiP)}}$$

Calculating the density of grafted chains on silica particles

The number of poly(VBC) chains per nm^2 was calculated by thermogravimetric analysis of the particles using Equation S2 where d is the grafting density, w is the percent mass lost on thermolysis, M_n is the molecular weight of the polymer, A_v is Avogadro's number, $\rho_{(SiP)}$ is the density of silica particles (1.9 x 10^{-21} $g{\cdot}nm^{-2}$), $V_{(SiP)}$ is the volume and $SA_{(SiP)}$ the surface area of a 130 nm silica particle.

Equation S2:

$$d = \frac{\frac{w_{(s)}}{100} \times M_n \times A_v}{\frac{\rho_{(SiP)}}{V_{(SiP)}} \times SA_{(SiP)}}$$

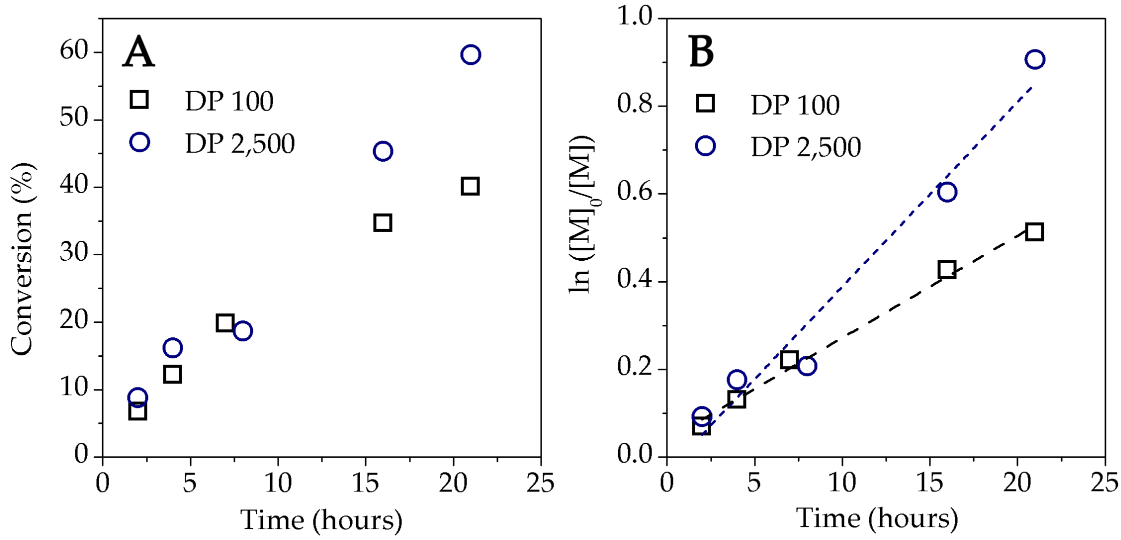

Figure S1: Conversion and ln([M]$_0$/[M]) vs time for thermally autoinitiated, PABTC-mediated polymerization of VBC. DP 100 (squares) and DP 2,500 (circles): A) monomer conversion vs reaction time; B) semi-log kinetics plots with dashed lines indicating linear fits of the data. (DP 100, long dashes; DP 2,500, short dashes).

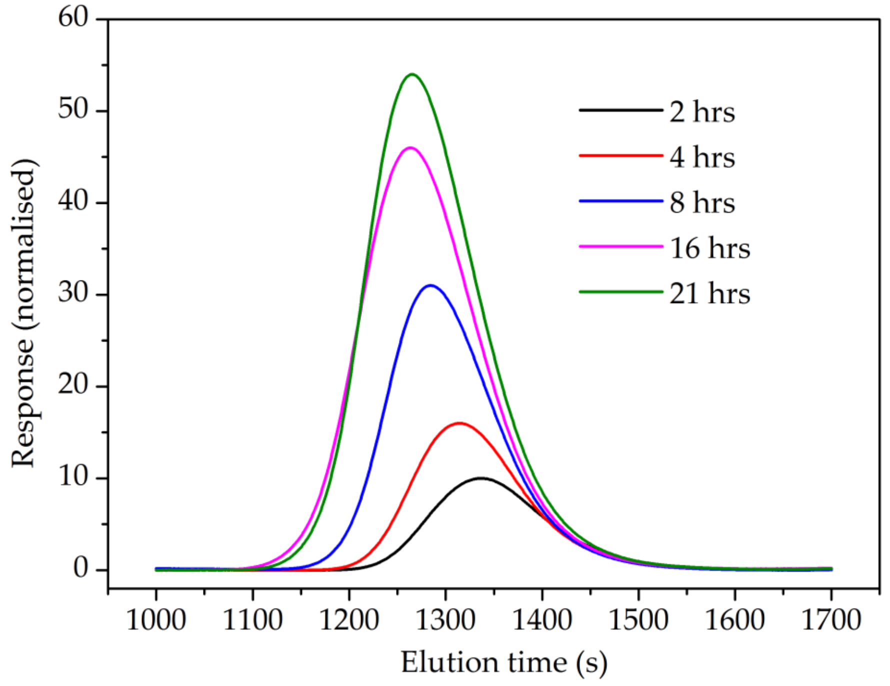

Figure S2: SEC traces of free polymers recovered from silica-supported RAFT polymerization of VBC at different conversions.

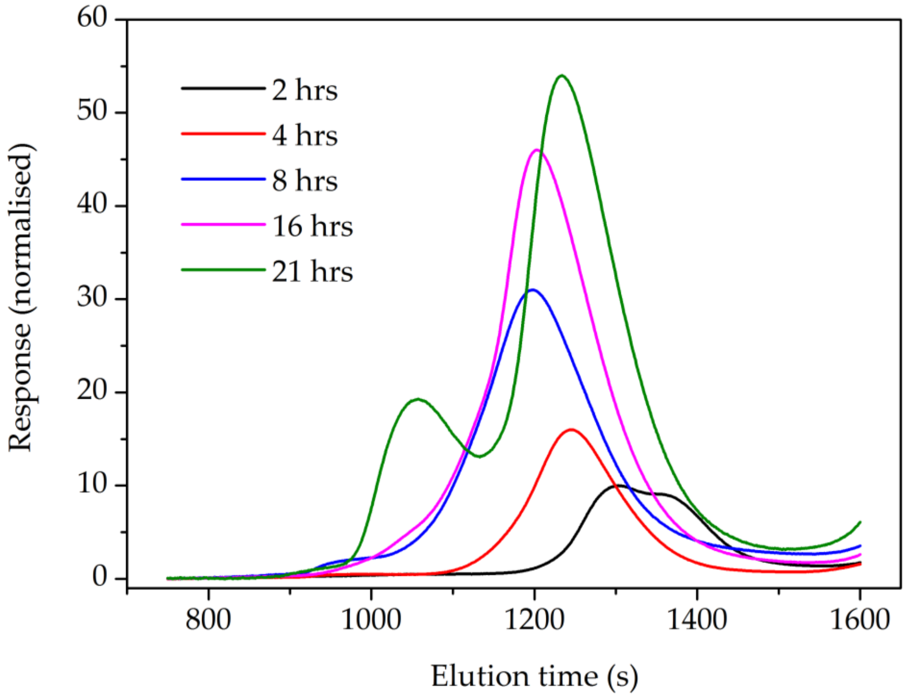

Figure S3: SEC traces of grafted polymers recovered from silica-supported RAFT polymerization of VBC at different conversions.

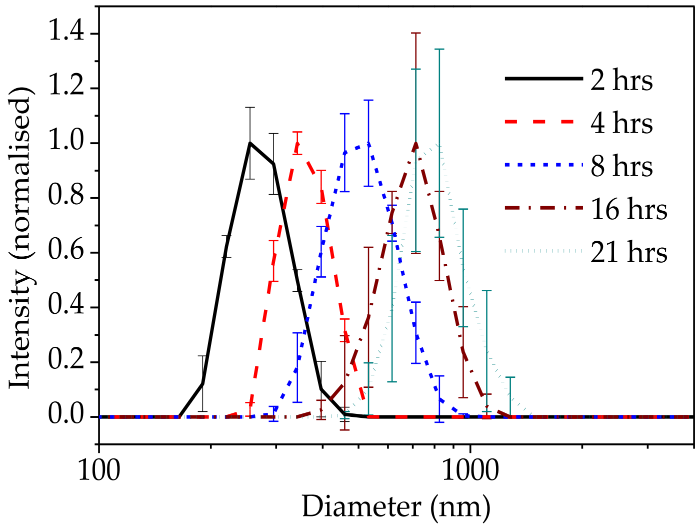

Figure S4: Particle-size distribution of particles recovered from silica-supported RAFT polymerization of VBC at different conversions.

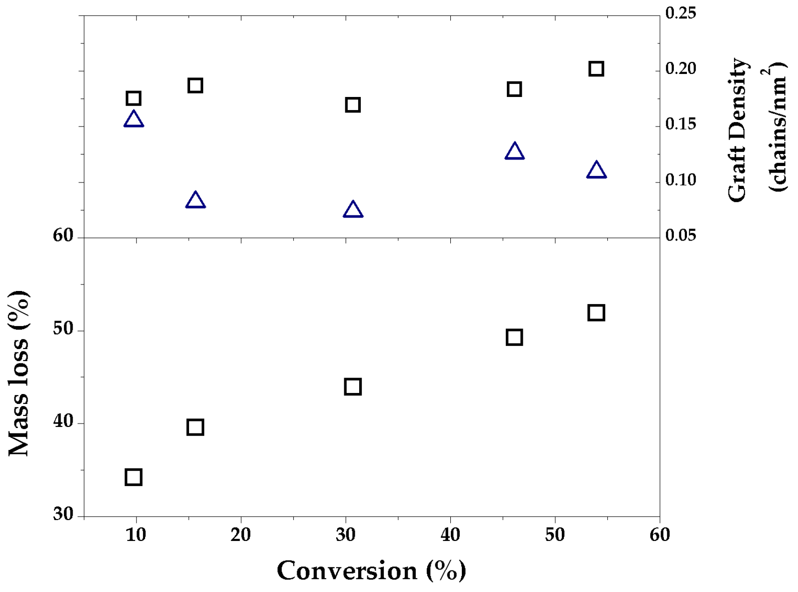

Figure S5: Plot of mass loss on thermolysis and grafting density of particles recovered from silica-supported RAFT polymerization of VBC. Squares represent grafting density calculated from M_n of free chains and triangles represent grafting density calculated from M_n of cleaved chains.

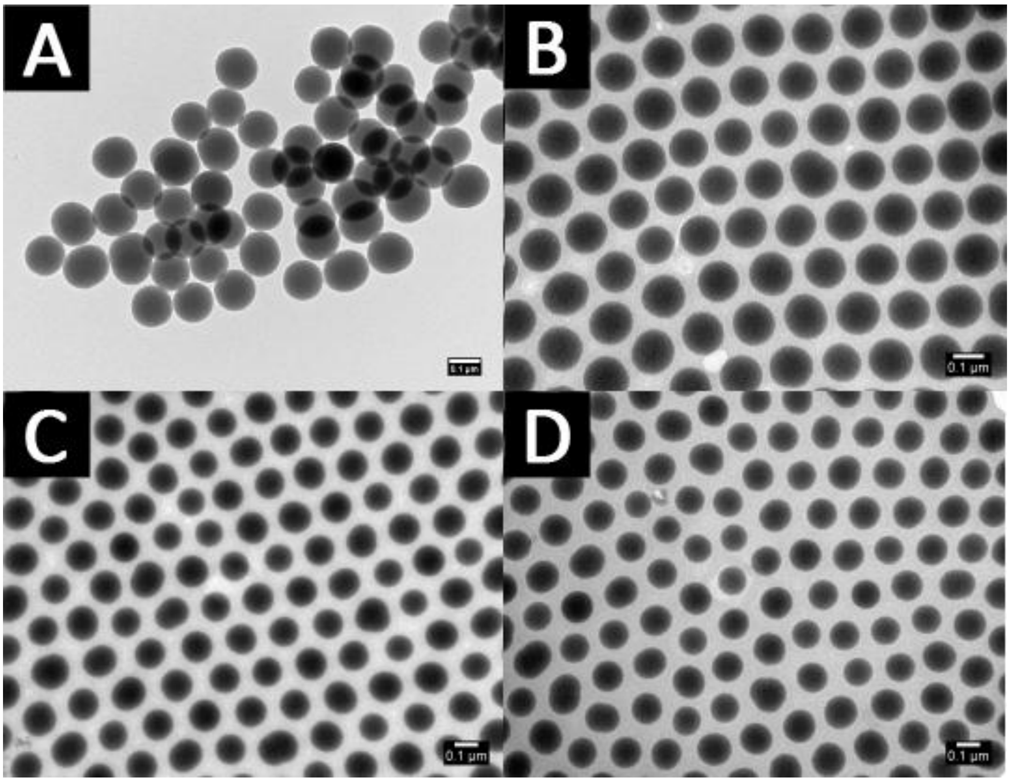

Figure S6: TEM micrographs of particles at various stages of VBC) polymerization: (A) before polymerization, (B) after 4 hours, (C) after 8 hours and (D) after 16 hours. Scale bars represent 0.1 μm.
